# Structural network alterations in adolescent major depression and bipolar disorder: a graph-theoretical and fixel-based analysis

**DOI:** 10.1186/s12888-026-07961-x

**Published:** 2026-03-10

**Authors:** Yijia Zhou, Yuhang Yang, Zixuan Cheng, Jingwen Liu, Yu Zhang, Jiaxin Luo, Xingyin Huang, Mingke Liu, Can Liu, Du Lei, Liangbo Hu

**Affiliations:** 1 Department of Radiology, The Affiliated Yongchuan Hospital of Chongqing Medical University, Chongqing, 402160 China; 2 College of Artificial Intelligence Medicine, Chongqing Medical University, Chongqing, 400016 China

**Keywords:** Major depressive disorder, Bipolar disorder, White matter, Graph theory, Fixel-based analysis

## Abstract

**Background:**

Major depressive disorder (MDD) and bipolar disorder (BD) commonly emerge during adolescence, a critical stage of brain development. Characterizing their shared and distinct neurobiological features is essential for early differential diagnosis.

**Methods:**

In this study, we investigated adolescents with major depressive disorder (MDD) (*n* = 48), bipolar disorder (BD) (*n* = 37), and healthy controls(*n* = 40). We acquired diffusion tensor imaging (DTI) data and applied a combined framework of graph-theoretical analysis and fixel-based analysis (FBA). We reconstructed structural networks using deterministic tractography and calculated global and nodal graph metrics. We then used FBA to measure fiber density (FD), fiber cross-section (FC), and combined FDC across major white matter tracts. We tested group differences and examined correlations with clinical severity after multiple-comparison correction.

**Results:**

Global graph metrics did not differ across groups, whereas nodal alterations were observed in regions assigned to the default mode, salience, and central executive networks, with disorder specific patterns. Fixel based analysis indicated widespread tract involvement in major depressive disorder but more circumscribed abnormalities in bipolar disorder, particularly in the corpus callosum and fornix. Several metrics were associated with symptom severity.

**Conclusions:**

These findings suggest disorder-related alterations in nodal topological properties and fiber-specific microstructure in adolescent MDD and BD, providing insight into the neurobiological basis of mood symptoms during this developmental stage. While these structural features may assist future efforts in differential characterization or individualized risk assessment, their potential clinical utility requires further validation.

**Clinical trial number:**

Not applicable.

**Supplementary Information:**

The online version contains supplementary material available at 10.1186/s12888-026-07961-x.

## Introduction

As disabling public health problems with high global prevalence, major depressive disorder (MDD) and bipolar disorder (BD) represent severe mood disorders whose neuropathological mechanisms remain unclear [1]. Adolescence is a critical developmental period during which the brain undergoes rapid and coordinated maturation, including increasing myelination, gray-matter reorganization, and refinement of large-scale functional and structural networks [2]. Importantly, both MDD and BD frequently emerge during this vulnerable period, disruptions to normative neurodevelopment may interfere with white matter and network maturation. Therefore, characterizing structural abnormalities in adolescents is essential for understanding the early neurobiological foundations of these disorders [3].

A growing body of neuroimaging research demonstrates that MDD and BD are associated with significant abnormalities in white matter architecture. These alterations prominently involve fronto-limbic and prefrontal–subcortical pathways that support emotional regulation [4, 5], as well as in major association tracts such as the superior longitudinal fasciculus (SLF), inferior longitudinal fasciculus (ILF), inferior fronto-occipital fasciculus (IFOF), and in interhemispheric commissural fibers of the corpus callosum [6–8]. Moreover, both disorders also involve widespread deficits in large-scale structural brain networks, including disrupted small-world organization, reduced global and local efficiency, and altered hub connectivity [9–11]. Furthermore, white matter microstructural and structural-connectivity alterations have been consistently associated with depressive symptom severity in adolescents with MDD and with mood-symptom burden in BD [6, 8, 12–14].

Whole-brain white matter networks are commonly analyzed using graph-theoretical approaches, sometimes together with network-based statistics (NBS), to assess topological properties such as nodal degree, nodal strength, and shortest path length [15, 16]. While these methods are useful for identifying network-level disruptions, NBS is optimized for edge-wise inference of connected components, requires spatially contiguous effects, and does not capture tract-specific microstructure or nodal topological roles [16, 17]. In addition, traditional diffusion tensor metrics such as fractional anisotropy (FA) and skeleton-based projections lack biological specificity and are particularly limited in regions with complex fiber configurations [18, 19]. Fixel-based analysis (FBA) offers a complementary framework that resolves multiple fiber populations within a voxel and quantifies fiber density (FD), fiber cross-section (FC), and their combined measure (FDC), enabling more precise characterization of microstructural and tract-level morphometric alterations, particularly in white matter regions with complex crossing-fiber architecture [20, 21], that undergo substantial remodeling during adolescence [22]. Integrating graph-theoretical indices with FBA-derived metrics therefore enables a multilevel characterization of white matter abnormalities, linking macro-scale network topology with tract-specific microstructural properties [23–25].

Therefore, this study applied a combined framework of graph-theoretical analysis and FBA to characterize structural network alterations in adolescents with MDD and BD, and to examine their clinical relevance. We hypothesized that MDD and BD would show both shared and disorder-specific alterations in white matter microstructure and structural network topology, and that some of these changes would be associated with symptom severity.

## Materials and methods

The Ethics Committee of Yongchuan Hospital Affiliated to Chongqing Medical University approved this cross-sectional study. The study protocol complies with the Declaration of Helsinki and has been approved by the local ethics committee. All participants and their parents or guardians received information about the study procedures and signed informed consent forms to participate. The study followed the STROBE (Strengthening the Reporting of Observational Studies in Epidemiology) reporting guidelines.

### Participants

Between January 2020 and December 2024, 48 adolescents with MDD and 37 adolescents with BD were consecutively recruited for this study through the Department of Psychiatry, The Affiliated Yongchuan Hospital of Chongqing Medical University, Chongqing, China. Healthy controls (HCs) were recruited from the general population through local media advertisements.

The inclusion criteria for patients with MDD were as follows: (a) diagnosis of MDD made by two trained psychiatrists using the Structured Clinical Interview for DSM-5 (SCID-5); (b) aged 12 to 19 years, right-handed; (c) the 24-item Hamilton Depression Rating Scale (HAMD-24) [26] score ≥ 20; (d) drug-naïve, defined as no psychotropic medication use prior to enrollment.

The inclusion criteria for patients with BD were as follows: (a) diagnosis of BD made by two trained psychiatrists using the Structured Clinical Interview for DSM-5 (SCID-5); (b) aged 12 to 19 years, right-handed; (c) assessed during a depressive episode, defined as HAMD-24 ≥ 20; (d) drug-naïve, defined as no psychotropic medication use prior to enrollment.

The inclusion criteria for the HCs were as follows: (a) no personal or family history of psychiatric disorders, confirmed by a psychiatrist using the SCID-5; (b) aged 12 to 19 years, right-handed.

Exclusion criteria for all participants were as follows: (a) history of severe medical, neurological, or psychiatric disorders; (b) substance use disorders, head trauma, or loss of consciousness; (c) any conditions unsuitable for MRI scanning.

The HAMD-24 and the Hamilton Anxiety Rating Scale (HAMA) [27] were used to evaluate the severity of depressive and anxiety symptoms, respectively. For both scales, higher scores indicate more severe symptoms.

### MRI data acquisition

All MRI data were collected on a Siemens 3.0T MRI with an 8-channel phased-array head/neck coil. The T1-weighted images were acquired using a three-dimensional magnetization-prepared rapid acquisition gradient echo (MPRAGE) sequence with the following parameters: 192 slices, repetition time (TR) = 2530 ms; echo time (TE) = 2.01 ms; field of view (FOV) = 256 × 256 mm; voxel size = 1 × 1 × 1 mm^3^. A total of 240 time points were obtained over 17 min. The parameters of diffusion−tensor imaging (DTI) images were as follows: TR = 11,200 ms, TE = 96.0 ms, acquisition matrix = 112 × 112, FOV = 224 mm × 224 mm, slice thickness = 2.0 mm, number of slices = 75, voxel resolution = 2.0 mm × 2.0 mm × 2.0 mm, flip angle = 90°, number of diffusion gradient directions = 64, b = 0 and 1000 s/mm2, number of excitations = 1.

### MRI preprocessing

The raw diffusion MRI data and T1-weighted structural images were converted from DICOM to NIfTI format using dcm2niix. A comprehensive preprocessing pipeline was implemented leveraging MRtrix3 (https://www.mrtrix.org/) and FSL (https://fsl.fmrib.ox.ac.uk) to ensure high data quality and anatomical accuracy. First, the diffusion data were denoised using MRtrix3’s dwidenoise [28] function, which implements the MP-PCA (Marchenko-Pastur Principal Component Analysis) method to enhance the signal-to-noise ratio. This was followed by Gibbs ringing [29] removal (mrdegibbs) using the local subvoxel-shift method to suppress artifacts near tissue boundaries. Subsequently, FSL’s eddy tool [30] was employed to correct for eddy current-induced distortions and subject head motion, performing outlier replacement and rotating the b-vectors accordingly. Bias field inhomogeneity was then corrected using the N4 algorithm [31]. For anatomical alignment, the T1-weighted structural images were skull-stripped and rigidly coregistered to the mean b0 diffusion image [32] (using a rigid-body transformation, 6 degrees of freedom) to ensure precise spatial correspondence between structural and diffusion modalities. Finally, a brain mask was generated to restrict subsequent analyses to brain tissue.

### Structural network analysis pipeline

Fiber tracking and network construction were performed using DSI Studio (https://dsi-studio.labsolver.org/) and GRETNA (https://www.nitrc.org/projects/gretna/) [33]. The preprocessed diffusion data were reconstructed using Q-Space Diffeomorphic Reconstruction (QSDR) [34], which calculates the spin distribution function (SDF) and normalizes the data to the standard MNI space. This spatial normalization facilitated the use of the Automated Anatomical Labeling (AAL) atlas to define network nodes. Whole-brain deterministic tractography was conducted with an angular threshold of 45° and a length range of 20–200 mm. The brain was parcellated into 90 ROIs (nodes) based on the AAL atlas, with the number of streamlines connecting each pair of regions serving as edge weights.

To strictly evaluate small-world topology and address potential confounds related to overall connectivity density, a comprehensive normalization procedure was applied. For each participant, 1000 degree-matched random networks were generated using the Maslov-Sneppen rewiring algorithm (preserving the number of nodes, edges, and degree distribution). Crucially, this algorithm generates null models that preserve the exact number of nodes, edges, and degree distribution of the original network. Small-world metrics (clustering coefficient (Cp) [35] and characteristic path length (Lp)) were normalized by the corresponding mean values of these 1000 random networks to derive normalized clustering coefficient (γ), normalized characteristic path length (λ), and the small-worldness scalar (σ). By benchmarking against null models with identical connectivity density, this normalization approach implicitly accounts for the influence of global connectivity strength on the topological characterization. Finally, nodal metrics including nodal degree, nodal betweenness, and nodal efficiency were calculated to assess regional network properties [15]. Detailed preprocessing steps are provided in the Supplementary Methods.

### Fixel-based analysis pipeline

FBA was performed using the Mrtrix3 official documentation pipeline [21, 36]. The preprocessing steps for DTI images included denoising [28], removal of Gibbs ring artifacts [29], correction for eddy current and motion-induced distortions, bias field correction, and upscaling the spatial resolution of DWI images to isotropic voxel sizes of 1.25 mm. We utilise group-average response functions for white matter, grey matter, and cerebrospinal fluid. Since the data is single-shell, we use MRtrix3Tissue (https://3Tissue.g.ithub.io) to estimate the FOD for each participant. The remaining processing steps included global image intensity normalisation of FOD images to enable FOD amplitude comparisons between participants using a median b = 0 intensity, and random selection of 10 FOD images from the three subgroups of participants to generate a study-specific FOD template 9, followed by linear and non-linear registration. Then, the study-specific population template is transformed into MNI152 public space using FA-based affine registration in the FMRIB Software Library 5.0.9 (FSL; Oxford Centre for Functional MRI of the Brain, UK; www.fmrib.ox.ac.uk/fsl). In simple terms, the white matter analysis template fixel mask is generated by segmenting the FOD into individual fixels. Using connectivity-based fixel enhancement, fixels within the mask are used during statistical analysis to identify the optimal fixel correspondence across all participants.

The following fixel-based metrics were calculated: (1) FD, which measures the fiber density of fiber bundles in each voxel unit. (2) FC, a morphological measurement of the cross-sectional size of fiber bundles, calculated by the degree of bundle cross-sectional distortion required to deform the participant’s FOD to the template image. (3) FDC, a combined measure of FD and FC (product), which can explain both microscopic and macroscopic changes in fiber bundles, thereby providing sensitivity to any differences related to white matter’s ability to transmit information.

### Tract of interest analysis

**Fig. 1:**
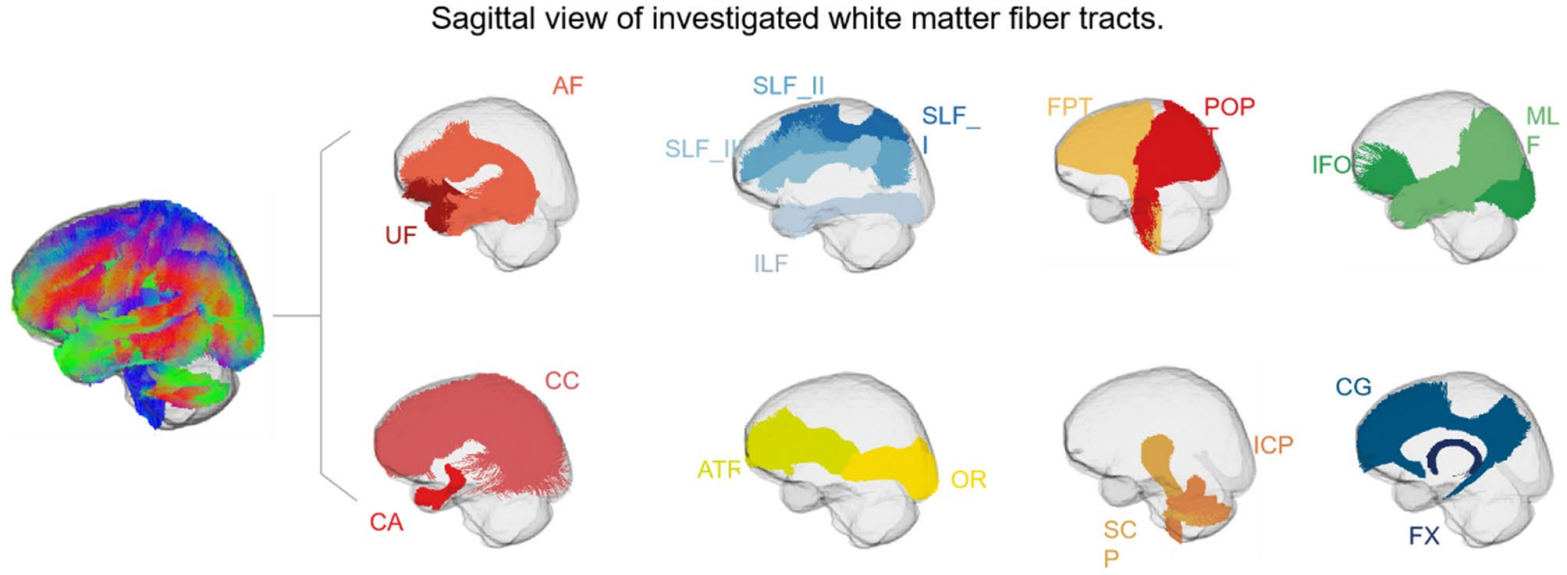
Sagittal view of investigated white matter fiber tracts. We selected 18 fiber bundles that were interpretable at the group level and associated with MDD and BD for fiber density (FD), fiber cross-section (FC), and fiber density and cross-section (FDC) analysis. AF = arcuate fasciculus; ATR = anterior thalamic radiation; CA = commissure anterior; CC = corpus callosum; CG = cingulum; MLF = middle longitudinal fasciculus; FPT = fronto-pontine tract; FX = fornix; ICP = inferior cerebellar peduncle; IFO = inferior occipito-frontal fasciculus; ILF = inferior longitudinal fasciculus; OR = optic radiation; POPT = parieto-occipital pontine; SCP = superior cerebellar peduncle; SLF_I = superior longitudinal fasciculus I; SLF_II = superior longitudinal fasciculus II; SLF_III = superior longitudinal fasciculus III; UF = uncinate fasciculus

ROI analysis was used to further explore potential degenerative changes in white matter fiber tracts in individuals with MDD and BD. TractSeg, a deep learning-based framework for automatic white matter tract segmentation, was used to segment the FOD template into 72 anatomically well-defined white matter fibers for further investigation of potential white matter degeneration in fiber tracts in individuals with MDD and BD. TractSeg was applied to study specific group templates, which were transformed into the MNI152 public space. We outlined the white matter tracts previously associated with individual groups of MDD or BD (Fig. 1) and provided interpretability at the group level. These include the arcuate fasciculus (AF), anterior thalamic radiation (ATR), anterior commissure (CA), corpus callosum (CC), cingulum (CG), middle longitudinal fascicle (MLF), fronto-pontine tract (FPT), inferior cerebellar peduncle (ICP), inferior occipito-frontal fascicle (IFO), inferior longitudinal fascicle (ILF), OR = optic radiation (OR), parieto-occipital pontine tract (POPT), superior cerebellar peduncle (SCP), superior longitudinal fascicle I (SLF_I), superior longitudinal fascicle II (SLF_II), superior longitudinal fascicle III (SLF_III), and uncinate fascicle (UF).

### Statistical analyses

Statistical analysis of demographic data was performed using IBM SPSS Statistics 27.0 (IBM Corp., Armonk, NY, USA). Demographic statistics and clinical scale scores were tested using a two-sample t-test. Gender differences between the two groups were analysed using a chi-square test. For brain network topological attribute data that did not follow a normal distribution, a non-parametric permutation test based on MATLAB (10,000 iterations) was used to identify significant differences in AUC values between groups. The 95th percentile of each distribution was used as the critical value for the two-tailed null hypothesis test, with a Type I error of 0.05. All analyses were adjusted for age and gender. Group differences in nodal attributes were assessed using FWE correction. For fiber tract metrics, generalized linear models were used to compare FD, FC, and FDC values between groups, with FWE correction applied. Associations with clinical scales were examined using partial correlation analyses in R (version 4.3.2).

## Results

### Demographic and clinical characteristics

A total of 125 adolescents successfully completed DTI scanning and were included in the analysis (HC = 40, MDD = 48, BD = 37). There were no significant differences between the three groups in age, BMI, and years of education (*P* > 0.05), but there were statistically significant differences in terms of gender (*P* < 0.05). The BD group showed numerically higher HAMD-24 and HAMA scores than the MDD group, but the differences were not statistically significant (Table 1).

**Table 1:** Demographic and clinical characteristics

Variables		HC (*n* = 40)	MDD (*n* = 48)	BD (*n* = 37)	*P*-value
Age		16.35 ± 1.79	15.65 ± 1.82	15.81 ± 1.47	0.403*
Sex (male/female)		20/20	11/37	11/26	0.017#
BMI		20.91 ± 2.56	20.6 ± 3.6	20.76 ± 3.26	0.078*
Education^a^ (years)		9.49 ± 2.15	9.40 ± 2.05	9.81 ± 1.47	0.706*
HAMD-24			34.33 ± 8.52	39.16 ± 9.32	0.412*
HAMA			28.65 ± 7.34	31.16 ± 7.71	0.416*

Data were represented as mean ± standard deviation unless otherwise indicated. * t-test; # chi-square test. Abbreviations: HC, healthy controls; MDD, Major Depressive Disorder; BD, Bipolar Disorder; HAMD-24,24-item Hamilton Depression Scale; HAMA, Hamilton Anxiety Rating Scale; ^a^ Education level represents the total number of completed years of formal schooling

### Nodal-level alterations in structural network topology

Although no significant group differences were observed in global topological metrics across MDD, BD, and HC, all three group comparisons showed significant nodal topological alterations, predominantly within the default mode network (DMN), salience network (SN), and central executive network (CEN). To complement the nodal findings, we also performed subnetwork-level analyses in the DMN, SN, and CEN by averaging nodal metrics within each network. At the subnetwork level, the DMN, SN, and CEN showed significant group differences in network-averaged nodal metrics (Supplementary Table S5).

#### Comparison of MDD vs. HC

**Fig. 2:**
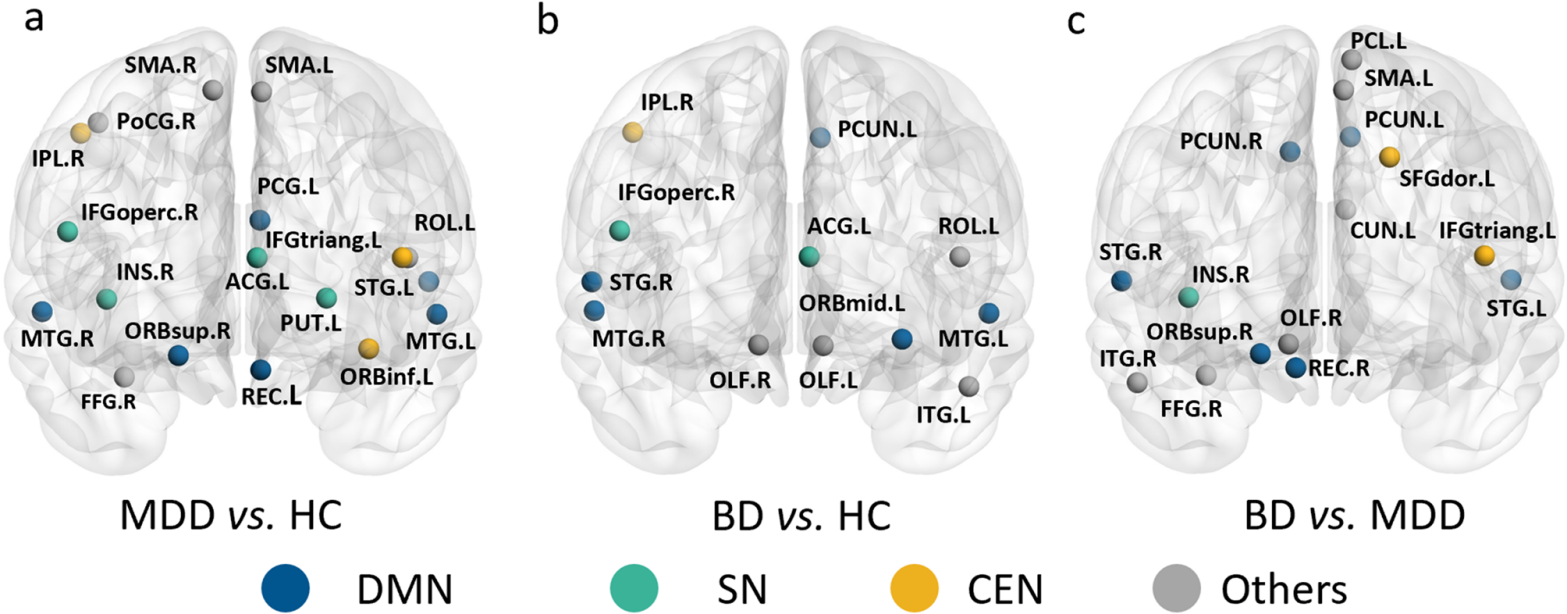
Group differences in efficiency, degree and betweenness at the nodal level. (**a**) MDD vs. HC, (**b**) BD vs. HC, (**c**) BD vs. MDD

Adolescents with MDD showed widespread nodal alterations across the SN, DMN, and CEN, with distinct patterns across regions assigned to canonical networks. Within the SN, the right INS, left PUT, and left ACC exhibited increased nodal metrics. Within the DMN, the left PCC, right ORBsup, and left STG showed increases, whereas the bilateral MTG showed decreases. Within the CEN, the left IFGorb and IFGtri showed decreases in nodal metrics (Table 2; Fig. 2a, Supplementary Table S1).

#### Comparison of BD vs. HC

Adolescents with BD showed a nodal pattern with a greater concentration of altered regions assigned to the DMN, accompanied by additional nodal changes in the SN and CEN. Within the DMN, the left PCUN and bilateral MTG exhibited increases in nodal metrics, whereas the right STG and left ORBmid showed decreases. Within the SN, the left ACC showed decreases, whereas the right IFGoper showed increases. Within the CEN, the right IPL exhibited decreases (Table 2; Fig. 2b, Supplementary Table S1).

#### Comparison of BD vs. MDD

Differences between BD and MDD were relatively focal, primarily involving DMN and CEN nodes. Within the DMN, compared with MDD, BD showed decreases in the bilateral PCUN, right REC, and bilateral STG, whereas the right ORBsup showed increases. Within the SN, the right INS showed decreases. Within the CEN, the left IFGtri showed increases, whereas the left SFG showed decreases (Table 2; Fig. 2c, Supplementary Table S1).

Across all significant nodal findings, effect sizes were in the moderate to large range (|d| ≈ 0.28–0.78).

**Table 2:** Brain regions with significant differences in nodal centralities across groups

Brain Regions	Nodal betweenness		Nodal degree		Nodal efficiency	
	*p*	Cohen’s d	*p*	Cohen’s d	*p*	Cohen’s d
MDD vs. HC						
Supp_Motor_Area_L	**0.027**↑	0.482	**0.009**↑	0.573	**0.006**↑	0.576
Supp_Motor_Area_R	**0.01**↑	0.564	**0.01**↑	0.564	**0.006**↑	0.604
Frontal_Inf_Orb_L	**0.009**↓	-0.573	**0.013**↓	-0.543	**0.017**↓	-0.521
Postcentral_R	**0.011**↑	0.557	**0.048**↑	0.430	0.006	0.604
Cingulum_Post_L	**0.07**	0.393	**0.04**↑	0.447	**0.043**↑	0.44
Frontal_Sup_Orb_R	**0.005**↑	0.617	**0.028**↑	0.479	**0.002**↑	0.683
Fusiform_R	**0.03**↓	-0.473	**0.03**↓	-0.473	**0.044**↓	-0.438
Rectus_L	**0.008**↑	0.582	**0.026**↑	0.485	0.05	0.426
Insula_R	**0.016**↑	0.526	**0.045**↑	0.436	0.1	0.356
Putamen_L	**0.017**↑	0.521	0.06	0.408	**0.031**↑	0.47
Temporal_Sup_L	**0.027**↑	0.482	**0.013**↑	0.543	**0.003**↑	0.655
Parietal_Inf_R	**0.015**↑	0.532	**0.003**↑	0.655	0.08	0.379
Frontal_Inf_Oper_R	0.21	0.270	**0.035**↑	0.459	**0.043**↑	0.44
Frontal_Inf_Tri_L	**0.003**↓	-0.612	**0.001**↓	-0.631	**0.003**↓	-0.655
Rolandic_Oper_L	**0.04**↓	-0.447	**0.007**↓	-0.592	**0.036**↓	-0.456
Temporal_Mid_L	**0.008**↓	-0.582	**0.001**↓	-0.734	**< 0.001**↓	-0.730
Temporal_Mid_R	**0.018**↓	-0.517	**0.021**↓	-0.504	**0.014**↓	-0.537
Cingulum_Ant_L	0.136	0.322	**0.027**↑	0.482	**0.019**↑	0.512
BD vs. HC						
Olfactory_L	**0.027**↑	0.515	**0.009**↑	0.612	**0.006**↑	0.536
Olfactory_R	**0.01**↑	0.534	**0.01**↑	0.604	**0.006**↑	0.646
Parietal_Inf_R	**0.04**↓	-0.477	**0.018**↓	-0.552	0.19	0.302
Frontal_Mid_Orb_L	**0.012**↓	-0.588	**0.005**↓	-0.571	**0.007**↓	-0.633
Frontal_Inf_Oper_R	0.05	0.455	**0.035**↑	0.490	**0.048**↑	0.459
Rolandic_Oper_L	**0.007**↓	-0.633	**< 0.001**↓	-0.619	**< 0.001**↓	-0.783
Temporal_Inf_L	0.08	0.405	**0.028**↑	0.512	**0.03**↑	0.505
Cingulum_Ant_L	0.225	0.279	**0.005**↓	-0.661	**0.001**↓	-0.783
Precuneus_L	**0.006**↑	0.646	**0.018**↑	0.457	**0.026**↑	0.519
Temporal_Sup_R	0.06	0.436	**0.011**↓	-0.595	**< 0.001**↓	-0.697
Temporal_Mid_L	0.07	0.42	**0.001**↑	0.773	**< 0.001**↑	0.756
Temporal_Mid_R	**< 0.001**↑	0.781	**0.006**↑	0.646	**0.007**↑	0.633
BD vs. MDD						
Frontal_Inf_Tri_L	**< 0.001**↑	0.721	**< 0.001**↑	0.747	**0.001**↑	0.724
Olfactory_R	**0.004**↑	0.648	**0.012**↑	0.562	**0.005**↑	0.628
Supp_Motor_Area_L	**0.038**↓	-0.462	**0.01**↓	-0.574	**0.008**↓	-0.595
Rectus_R	**0.011**↓	-0.570	**0.023**↓	-0.507	**0.024**↓	-0.503
Frontal_Sup_L	**0.001**↓	-0.718	**0.011**↓	-0.549	**0.005**↓	-0.623
Insula_R	**< 0.001**↓	-0.728	**0.005**↓	-0.632	**0.002**↓	-0.699
Cuneus_L	**0.006**↑	0.618	**0.011**↑	0.526	**0.01**↑	0.557
Fusiform_R	**0.006**↓	-0.618	**0.012**↓	-0.562	**0.01**↓	-0.541
Frontal_Sup_Orb_R	**0.02**↑	0.519	**0.004**↑	0.648	**0.002**↑	0.699
Precuneus_L	**< 0.001**↓	-0.720	**0.002**↓	-0.699	**0.002**↓	-0.674
Precuneus_R	**< 0.001**↓	-0.719	**0.01**↓	-0.523	**0.01**↓	-0.577
Temporal_Sup_L	0.097	-0.367	**0.01**↓	-0.519	**0.018**↓	-0.528
Temporal_Sup_R	0.092	-0.373	**< 0.001**↓	-0.736	**< 0.001**↓	-0.737
Temporal_Inf_R	0.029	0.486	**0.03**↑	0.483	**0.017**↑	0.533
Paracentral_Lobule_L	0.18	0.296	**0.026**↑	0.496	**0.036**↑	0.467

If these regions showed significant between-group differences in the MDD group or BD group, they were considered abnormal. At least two of the three nodes show differences (p < 0.05) (shown in bold). Cohen’ s d values represent standardized effect sizes. All brain regions are derived from automatic anatomical labelling (AAL). Abbreviations: MDD, major depressive disorder; HC, healthy controls; BD, Bipolar Disorder; L, left; R, right; ACC = anterior cingulate cortex; CEN = central executive network; DMN = default mode network; IFGoper = inferior frontal gyrus, opercular part; IFGorb = inferior frontal gyrus, orbital part; IFGtri = inferior frontal gyrus, triangular part; INS = insula; IPL = inferior parietal lobule; MTG = middle temporal gyrus; ORBmid = middle orbital frontal gyrus; ORBsup = superior orbital frontal gyrus; PCC = posterior cingulate cortex; PCUN = precuneus; PUT = putamen; REC = gyrus rectus; SFG = superior frontal gyrus; SN = salience network; STG = superior temporal gyrus

### White matter microstructural alterations

#### Comparison of MDD vs. HC

**Fig. 3:**
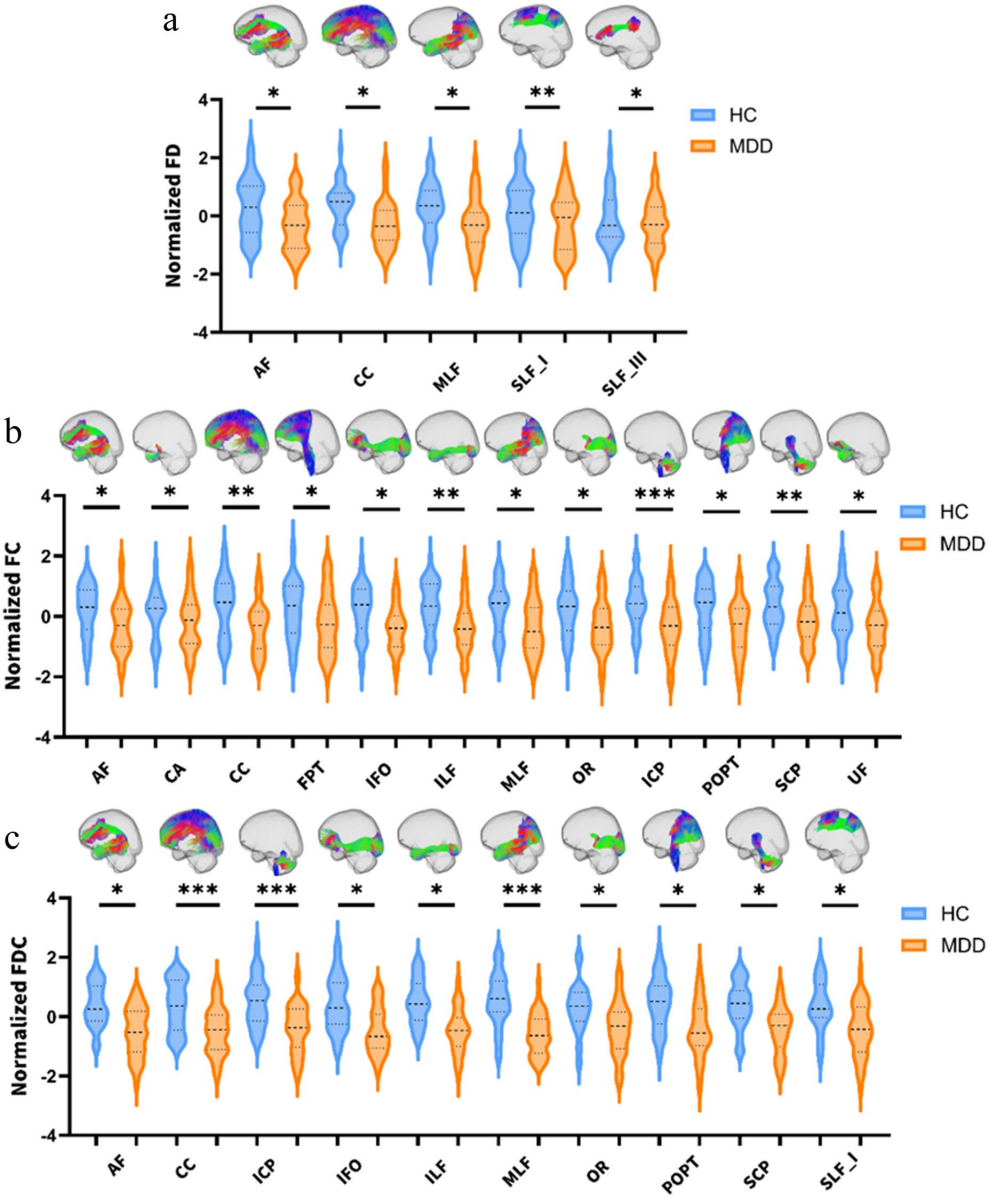

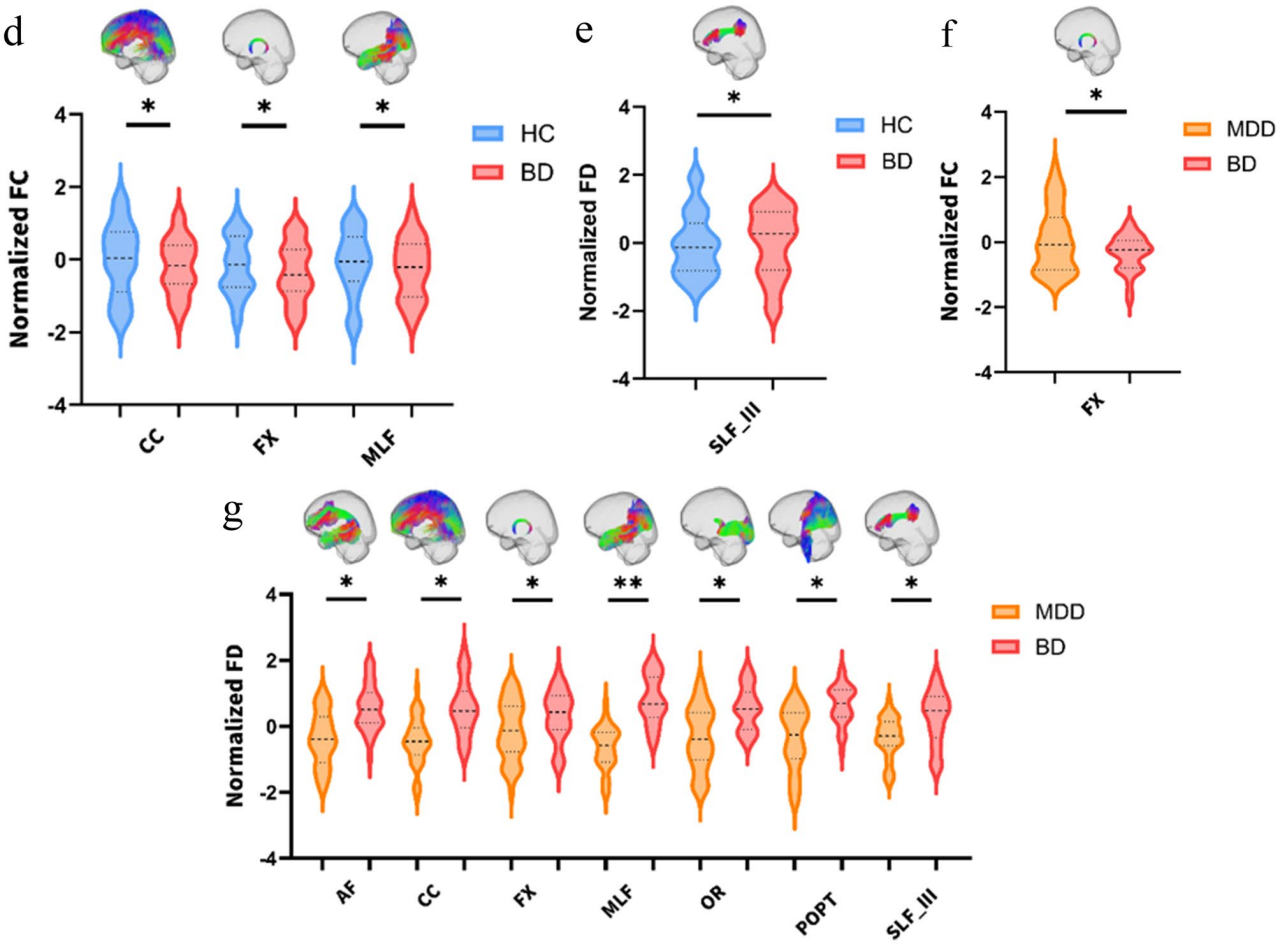
Group comparisons of white matter fixel-based metrics: (**a**) Fiber density (FD) differences between adolescents with MDD and HC. (**b**) Fiber cross-section (FC) differences between MDD and HC. (**c**) Combined fiber density and cross-section (FDC) differences between MDD and HC. (**d**) FC differences between BD and HC. (**e**) FD differences between BD and HC. (**f**) FC differences between BD and MDD. (**g**) FD differences between BD and MDD. Results are shown at *p* < 0.05, FEW-corrected. Statistical significance is indicated as *p* < 0.05 (*), *p* < 0.01 (**), and *p* < 0.001 (***)

The MDD group exhibited widespread reductions in FD, FC, and FDC across multiple white matter tracts. Specifically, significant FD reductions were observed in five tracts: AF, CC, MLF, SLF I, and SLF III (all FWE-corrected). FC reductions were identified across 12 white matter bundles, for example ICP, CC, ILF and SCP (all FWE-corrected). Similarly, FDC showed significant reductions in 10 tracts (all FWE-corrected) (Fig. 3a-c, Supplementary Table S2).

#### Comparison of BD vs. HC

The BD group showed more focal microstructural alterations compared with HCs. For FC, reductions were restricted to three tracts: CC, FX and MLF (all FWE-corrected). FD was increased in SLF III (FWE-corrected). No significant group differences were observed in FDC (Fig. 3d-e, Supplementary Table S3).

#### Comparison of BD vs. MDD

The BD group exhibited higher FD and FC values than the MDD group in several tracts. For FC, the BD group showed higher values in FX than MDD (FWE-corrected). For FD, the BD group exhibited higher values in 7 tracts, for example AF, CC, FX and MLF (all FWE-corrected). No significant group differences were observed in FDC (Fig. 3f-g, Supplementary Table S4).

Across all significant fixel-based differences between MDD and BD, most Cohen’s d values ranged from 0.20 to 0.50(overall range: 0.10–0.50), indicating overall small to moderate effect sizes.

### Symptom correlation results

#### Correlation between network topological properties and clinical ratings

**Fig. 4:**
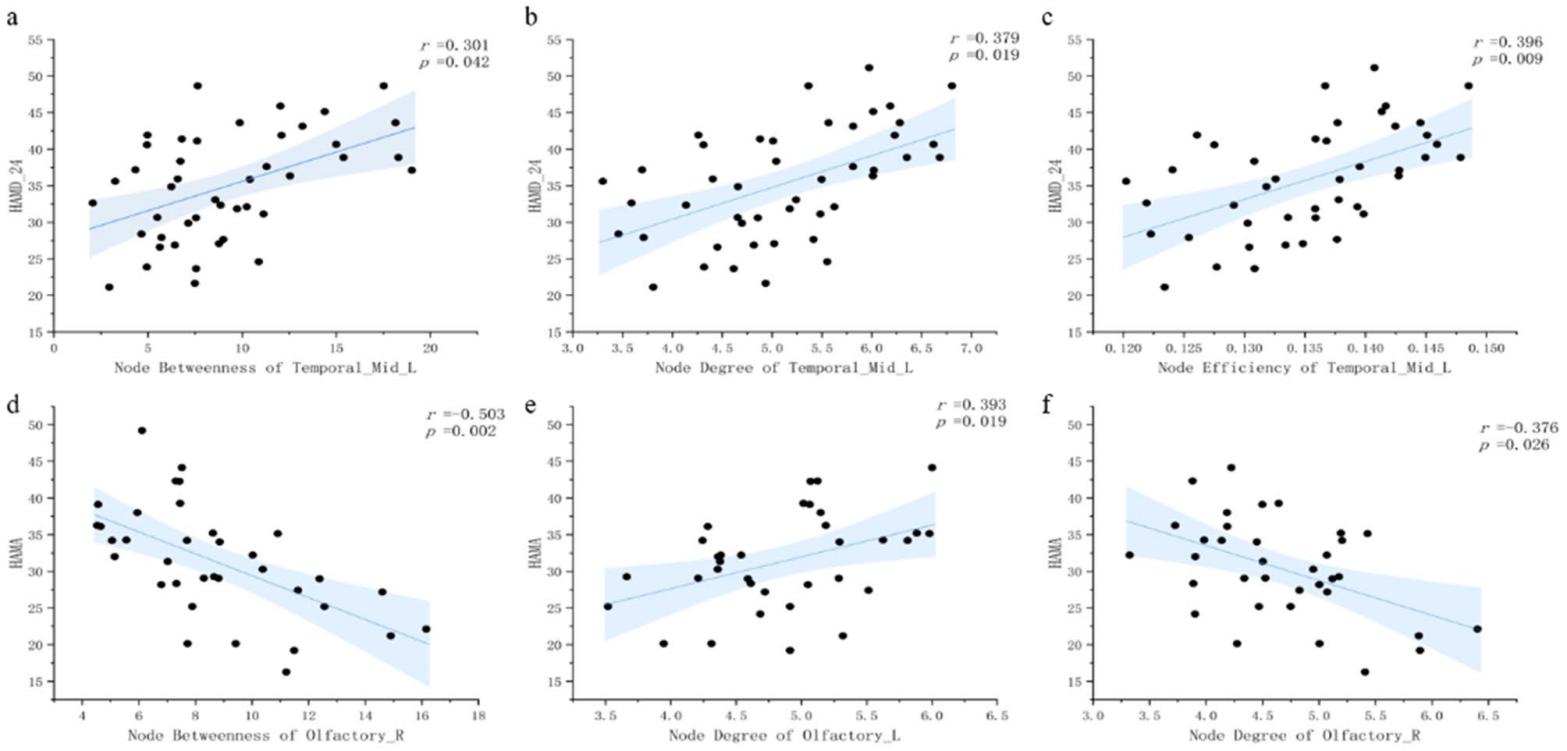
Correlation between topological properties and symptom severity. Node betweenness, nodal degree, and nodal efficiency of the left middle temporal gyrus and HAMD-24 scores (**a**, **b**, **c**). Nodal betweenness of the right olfactory cortex and HAMA scores (**d**). Nodal degree of the left and right olfactory cortex and HAMA scores (**e**, **f**)

In the MDD group, nodal betweenness, degree and efficiency of the left MTG were positively correlated with the HAMD-24 scores. In the BD group, the node degree and betweenness of the right OLF were negatively correlated with the HAMA scores. The node degree of the left OLF was positively correlated with the HAMA scores (Fig. 4).

#### Fiber bundle indices and clinical ratings

**Fig. 5:**
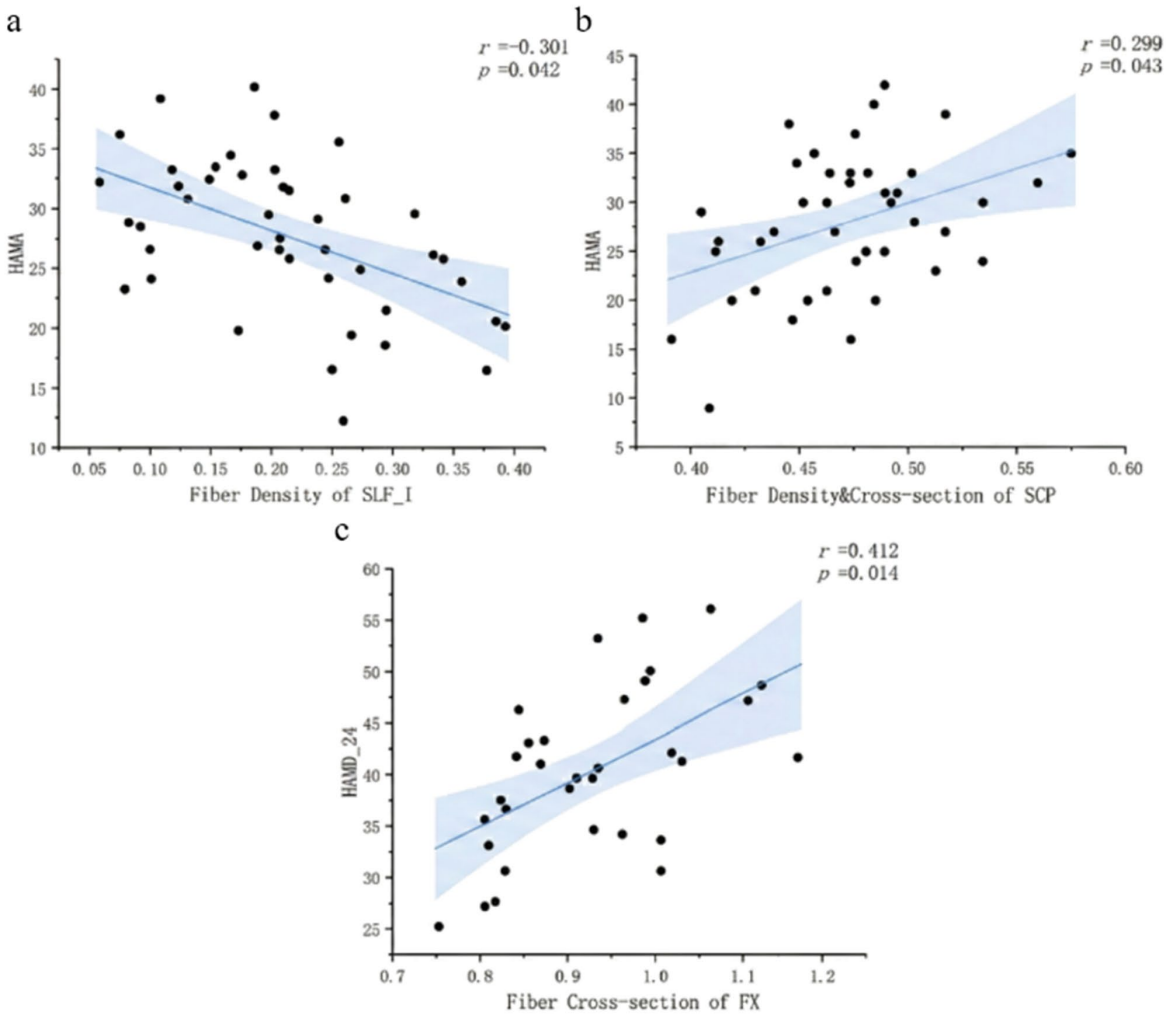
Correlation between white matter fiber tracts and symptom severity. FD of the superior longitudinal fasciculus branch I and HAMA scores (**a**). FDC of the superior cerebellar peduncle and HAMA scores (**b**). FC of the fornix and HAMD-24 scores (**c**)

In the MDD group, FD in SLF I was negatively correlated with HAMA scores. By contrast, FDC in SCP was positively correlated with HAMA scores. In the BD group, FC in FX was positively correlated with HAMD-24 scores (Fig. 5).

## Discussion

In this study, we combined graph-theoretical analysis of structural connectomes with FBA to characterize structural network topology and fiber-specific white matter alterations in drug-naïve adolescents with MDD and BD. We reported three key findings: (1) global topological metrics were did not differ across groups, whereas significant nodal-level alterations were observed, predominantly in nodes regions assigned to the DMN, SN, and CEN; (2) FBA indicated more extensive white matter microstructural alterations in MDD but a more limited pattern in BD; (3) several nodal metrics and FBA metrics showed modest associations with symptom severity. Collectively, these findings highlight shared and disorder-specific structural features in adolescent mood disorders and may inform future work on differential characterization, pending replication and validation in larger, longitudinal cohorts.

### Altered nodal metrics of the structural network

In this study, we observed no significant group differences in global graph metrics, consistent with prior reports indicating that nodal alterations can emerge despite relatively preserved global topology [37–39]. We identified nodal-level alterations in key regions assigned to the DMN, SN, and CEN in both MDD and BD. This nodal pattern is consistent with the complementary subnetwork-averaged analysis, suggesting that the DMN/SN/CEN-assigned effects are unlikely to be solely driven by the relative distribution of significant nodes.

In MDD, nodal-level topological alterations were distributed across multiple regions, with prominent increases in DMN- and SN-assigned regions, alongside region-specific decreases within the CEN. Notably, MDD showed increased degree and efficiency in SN-assigned regions, including the right INS, left PUT, and left ACC. This pattern is broadly consistent with prior work implicating the INS and ACC in altered salience detection and interoceptive processing in depression [40]. The insula is a key hub for integrating interoceptive and affective signals, and converging evidence links insular functional alterations to negative affective bias and increased processing of bodily signals in MDD [41–45]. Accordingly, the increased nodal centrality observed in the insula may represent a structural network correlate of altered salience and interoceptive signaling in adolescent depression, although its clinical relevance requires validation using multimodal and longitudinal designs. Further, consistent with prior reports of increased centrality or efficiency of DMN hubs in MDD, particularly involving the PCC and lateral temporal regions [46, 47], we observed increased nodal topological properties across most regions assigned to the DMN in our study. Taken together, increased nodal efficiency in these DMN-assigned regions may reflect increased topological prominence of DMN-related nodes within the structural network [48], aligning with functional models that link DMN-related processes to ruminative thinking and emotion regulation difficulties in MDD [49]. In contrast, within the CEN, the left IFGorb and IFGtri showed convergent reductions across nodal metrics. These reductions may suggest a diminished topological contribution of lateral prefrontal control systems within the structural network. The left IFG is also implicated in higher-order language related processes [50], and prior functional work has reported altered IFG engagement during emotional and cognitive processing in MDD [51–53]. Collectively, with concomitant increases in DMN- and SN-assigned regions, this pattern may be compatible with reduced top-down control in adolescent MDD.

A comparatively more focal pattern emerged in BD, with nodal alterations preferentially involving DMN-assigned regions, accompanied by more limited changes in SN- and CEN-assigned nodes. Adolescence represents a sensitive window for continued consolidation of default mode network architecture, with ongoing developmental changes in topological organization and efficiency [54, 55]. Accordingly, the preferential concentration of nodal alterations in DMN-assigned regions in BD may be compatible with this developmental sensitivity.

Notably, MTG and ACC showed opposite directional shifts relative to HCs across disorders, with MTG tending to increase in BD but decrease in MDD and the reverse pattern for the ACC. However, these regions did not show significant differences in the direct BD versus MDD comparison, indicating that the opposite directions should be interpreted as disorder-related deviations from HCs rather than robust diagnosis discriminating effects. This pattern may reflect limited effect sizes and substantial within group heterogeneity, and warrants replication in larger longitudinal samples. The direct BD versus MDD comparison further indicated that their nodal topological differences were not confined to a single network but involved multiple regions assigned to the DMN, CEN, and SN.

### Fixel-based analysis

FBA revealed fiber-specific white matter microstructural alterations in adolescents with MDD and BD relative to HCs, with more widespread tract involvement in MDD but a more limited pattern in BD. Overall, our tract level findings converge with prior diffusion studies across mood disorders that repeatedly implicate long-range association tracts and commissural tracts [56–62].

Notably, in our drug-naïve adolescents, we additionally observed abnormalities in projection and cerebellar tracts in MDD. Within projection fibers, OR and POPT showed reduced microstructural integrity. The OR is associated with semantic fluency [63], and the POPT plays a crucial role in visual information processing [64]. Microstructural abnormalities in the POPT may help explain the visuospatial and visuomotor deficits previously observed in mood disorders [56]. SCP and ICP are key cerebellar white matter tracts [65], and accumulating evidence implicates cerebellar circuit alterations in psychiatric disorders [66]. Prior morphometric and diffusion studies have reported cerebellar abnormalities in mood disorders, including reduced anterior cerebellar gray-matter volume and reduced fractional anisotropy and connectivity within cerebral networks [67, 68]. In the present study, SCP and ICP abnormalities in MDD provide further anatomical evidence that cerebellar tracts may be involved in adolescent depression [69].

In BD, the most notable finding was reduced FD and FC in the FX relative to HCs, consistent with large-scale evidence suggesting prominent fornix involvement in BD [70]. The FX is widely regarded as a major hippocampal efferent pathway within the Papez circuit, reduced FX FD and FC may indicate compromised hippocampal efferent integrity [71, 72]. We also observed reduced CC FD in BD. Both CC and FX have been implicated in working memory, problem solving, and declarative memory [73]. Cognitive impairments are common in both disorders, but BD is associated with more severe deficits in attention, memory, and executive function, especially in early-onset cases [74, 75]. Accordingly, reduced FX and CC microstructural integrity may relate to these cognitive difficulties. Finally, BD showed a directionally distinct increase in SLF-III FD. FD reflects the density of white matter, with higher values indicating increased intra-axonal volume [36], but such increases are not specific and may reflect heterogeneous factors, warranting cautious interpretation, potentially related to a heightened state of neuroinflammation and increased extra-axonal space filling with extracellular materials.

### Correlation analysis

Whereas prior studies in MDD have reported reduced topological measures in temporal cortical regions [76], we found that higher symptom severity was positively associated with increased nodal degree, betweenness, and efficiency in the left MTG. Given that nodal degree reflects the number of connections, nodal efficiency captures shortest path based communication efficiency and is commonly interpreted as a marker of topological integration, and betweenness centrality reflects a node’s potential influence on information flow [15], this pattern may suggest greater MTG involvement in structural network communication with increasing symptom burden. These associations are broadly compatible with rumination related models in depression [77–80]. Additionally, in BD, the right OLF nodal betweenness and degree were negatively correlated with symptom severity, whereas left OLF nodal degree was positively correlated with symptom severity, indicating a lateralized association with clinical severity [81]. The OLF is closely linked with the limbic system [82], and contemporary models of BD commonly implicate dysregulation of limbic and emotion regulatory systems [83]. Collectively, these results suggest that olfactory related limbic pathways may contribute to interindividual variability in anxiety symptom expression in BD, although the functional meaning of these structural nodal associations remains indirect and warrants replication and multimodal validation. Together, these findings emphasize that, beyond the classical prefrontal–limbic pathways, olfactory–emotional circuits may also contribute to the clinical expression of mood symptoms in BD.

We further analyzed how microstructural metrics relate to symptom severity. In BD, patients with severe symptom severity showed higher FC in the FX. In MDD, greater symptom severity was associated with lower FD in the SLF-I and higher FDC in the SCP. These results suggest potential structure–symptom coupling across hippocampal–limbic, association, and cerebellar pathways, but their biological interpretation is inherently non-specific. Lower FD indicates microstructural pathology linked to neuroinflammation or axonal injury [84], whereas increases in FC or FDC may reflect heterogeneous factors, some studies report that inflammation, gliosis, or partial volume effects may account for such changes [21, 84].

Overall, these exploratory associations indicate that symptom severity may relate to both nodal topology and fixel-derived measures in adolescent mood disorders, warranting replication in larger samples and longitudinal designs to clarify their stability, specificity, and potential predictive value.

### Limitations and future directions

This study has several limitations. First, the cross-sectional design and relatively modest sample size limit causal inference. Although several diffusion MRI based structural features were associated with depressive severity, these correlations do not establish temporal directionality or causality, and their clinical relevance remains preliminary. In addition, because the study relied solely on structural MRI and fixel-based analyses, the extent to which these structural alterations correspond to functional or metabolic changes remains unclear. Crucially, the modest effect sizes of the observed neuroimaging alterations underscore that the translational potential of these metrics as standalone biomarkers for individualized diagnosis is currently limited. Future large-sample, multi-center longitudinal studies incorporating multimodal imaging and integrative analytical approaches, including machine learning, are needed to clarify structure–function coupling, establish the robustness of these structural features, and better evaluate their potential translational value.

Second, the sex distribution across groups was uneven, which may introduce residual confounding because sex differences have a well-established impact on adolescent white matter development and structural network organization. Prior neurodevelopmental studies have shown that males and females follow distinct maturation trajectories in white matter tracts and network topology [85–87]. Although sex was included as a covariate, future studies with more balanced samples are required to confirm the robustness of our findings.

Third, although the AAL template has been widely used to construct white matter structural networks [88, 89], the AAL-90 parcellation has known limitations related to anatomical granularity and boundary definitions [90]. Future studies may benefit from adopting higher-resolution or multimodal parcellation, such as the Human Connectome Project multimodal atlas [91] or the Human Brain Network Atlas [92], to construct networks and obtain finer-grained network features.

## Conclusion

This study identified disorder-related alterations in nodal topological properties and fiber-specific microstructure in adolescent MDD and BD. Both disorders showed nodal-level abnormalities involving regions located within the DMN, CEN, and SN, and MDD additionally exhibited more extensive fiber-specific alterations compared with BD. Several of these features were associated with depressive severity, potentially informing the pathophysiology of adolescent mood disorders. These findings provide preliminary structural markers that may assist future research on differential characterization or individualized risk assessment, although their clinical applicability requires rigorous validation.

## Supplementary Information

Below is the link to the electronic supplementary material.

**Supplementary Material 1**

**Supplementary Material 2**: Supplementary Table S1. Brain regions with altered nodal centralities among HC, MDD, and BD

**Supplementary Material 3**: Supplementary Table S2. Differences in FBA measures between patients with MDD and HC

**Supplementary Material 4**: Supplementary Table S3. Differences in FBA measures between patients with BD and HC

**Supplementary Material 5**: Supplementary Table S4. Differences in FBA measures between patients with MDD and BD

**Supplementary Material 6**: Supplementary Table S5. Network-level nodal metrics in the DMN, SN, and CEN

## Data Availability

This article contains all the data generated or analyzed during this study. For any further inquiries, please contact the corresponding author.
