# Supplementary material for: Structural network alterations in adolescent major depression and bipolar disorder: a graph-theoretical and fixel-based analysis
Source: BMC Psychiatry. 2026 Mar 10;26:322. doi: 10.1186/s12888-026-07961-x (PMC13085478; doi:10.1186/s12888-026-07961-x)
Supplement: Supplementary file 1 — Supplementary Material 1 [file 12888_2026_7961_MOESM1_ESM.docx]

**Subnetwork Statistical Analysis**

All statistical analyses were performed in R (version 4.3.2). For each graph-theoretical nodal metric (nodal efficiency, degree, and betweenness), Network assignments were based on three canonical networks: the default mode network (DMN), salience network (SN), and central executive network (CEN). Network-level measures were then computed as the mean across all nodes within each network^[1,2]^. Group differences among HC, MDD, and BD were tested using analysis of covariance (ANCOVA), with the network-level metric as the dependent variable, group as the fixed factor, and age, sex, and years of education as covariates. The main effect of group was assessed using Type III sums-of-squares tests. For metrics with a significant group effect, post-hoc pairwise comparisons were conducted using estimated marginal means (EMMs). Statistical significance was set at two-tailed P < 0.05.

**References:**

[1] Vriend C, Van Den Heuvel O A, Berendse H W, et al. Global and subnetwork changes of the structural connectome in de novo parkinson’s disease[J]. Neuroscience, 2018, 386: 295-308.

[2] Keerativittayayut R, Aoki R, Sarabi M T, et al. Large-scale network integration in the human brain tracks temporal fluctuations in memory encoding performance[J]. eLife, 2018, 7: e32696.
