# Supplementary material for: Structural network alterations in adolescent major depression and bipolar disorder: a graph-theoretical and fixel-based analysis
Source: BMC Psychiatry. 2026 Mar 10;26:322. doi: 10.1186/s12888-026-07961-x (PMC13085478; doi:10.1186/s12888-026-07961-x)
Supplement: Supplementary file 2 — Supplementary Material 2: Supplementary Table S1. Brain regions with altered nodal centralities among HC, MDD, and BD [file 12888_2026_7961_MOESM2_ESM.docx]

**Supplementary Table S1. Brain Regions with Altered Nodal Centralities among HC, MDD, and BD: Pairwise Statistical Comparisons with t-values, Cohen’s d, and p-values.**

| Brain regions | Nodal degree | | | Nodal efficiency | | | Nodal betweenness | | |
| --- | --- | --- | --- | --- | --- | --- | --- | --- | --- |
|  | t | Cohen’s d | *p* value | t | Cohen’s d | *p* value | t | Cohen’s d | *p* value |
| MDD *vs.* HC |  |  |  |  |  |  |  |  |  |
| Supp_Motor_Area_L | 2.675 | 0.573 | 0.009 | 2.524 | 0.576 | 0.006 | 2.251 | 0.482 | 0.027 |
| Supp_Motor_Area_R | 2.636 | 0.564 | 0.010 | 2.823 | 0.604 | 0.006 | 2.636 | 0.564 | 0.010 |
| Frontal_Inf_Orb_L | -2.539 | -0.543 | 0.013 | -2.436 | -0.521 | 0.017 | -2.675 | -0.573 | 0.009 |
| Postcentral_R | 2.007 | 0.430 | 0.048 | 2.820 | 0.604 | 0.006 | 2.601 | 0.557 | 0.011 |
| Cingulum_Post_L | 2.087 | 0.447 | 0.040 | 2.055 | 0.44 | 0.043 | 1.836 | 0.393 | 0.070 |
| Frontal_Sup_Orb_R | 2.237 | 0.479 | 0.028 | 3.191 | 0.683 | 0.002 | 2.884 | 0.617 | 0.005 |
| Fusiform_R | -2.208 | -0.473 | 0.030 | -2.045 | -0.438 | 0.044 | -2.208 | -0.473 | 0.030 |
| Rectus_L | 2.267 | 0.485 | 0.026 | 1.989 | 0.426 | 0.050 | 2.718 | 0.582 | 0.008 |
| Insula_R | 2.035 | 0.436 | 0.045 | 1.663 | 0.356 | 0.100 | 2.459 | 0.526 | 0.016 |
| Putamen_L | 1.907 | 0.408 | 0.060 | 2.194 | 0.47 | 0.031 | 2.436 | 0.521 | 0.017 |
| Temporal_Sup_L | 2.539 | 0.543 | 0.013 | 3.058 | 0.655 | 0.003 | 2.251 | 0.482 | 0.027 |
| Parietal_Inf_R | 3.058 | 0.655 | 0.003 | 1.772 | 0.379 | 0.080 | 2.484 | 0.532 | 0.015 |
| Frontal_Inf_Oper_R | 2.143 | 0.459 | 0.035 | 2.055 | 0.44 | 0.043 | 1.263 | 0.270 | 0.210 |
| Frontal_Inf_Tri_L | -3.264 | -0.631 | 0.001 | -3.058 | -0.655 | 0.003 | -2.784 | -0.612 | 0.003 |
| Rolandic_Oper_L | -2.766 | -0.592 | 0.007 | -2.132 | -0.456 | 0.036 | -2.087 | -0.447 | 0.040 |
| Temporal_Mid_L | -3.412 | -0.734 | 0.001 | -3.412 | -0.730 | <0.001 | -2.718 | -0.582 | 0.008 |
| Temporal_Mid_R | -2.353 | -0.504 | 0.021 | -2.51 | -0.537 | 0.014 | -2.414 | -0.517 | 0.018 |
| Cingulum_Ant_L | 2.251 | 0.482 | 0.027 | 2.392 | 0.512 | 0.019 | 1.505 | 0.322 | 0.136 |
| BD *vs.* HC |  |  |  |  |  |  |  |  |  |
| Olfactory_L | 2.685 | 0.612 | 0.009 | 2.642 | 0.536 | 0.006 | 2.258 | 0.515 | 0.027 |
| Olfactory_R | 2.646 | 0.604 | 0.010 | 2.832 | 0.646 | 0.006 | 2.326 | 0.534 | 0.010 |
| Parietal_Inf_R | -2.421 | -0.552 | 0.018 | 1.323 | 0.302 | 0.190 | -2.092 | -0.477 | 0.040 |
| Frontal_Mid_Orb_L | -2.474 | -0.571 | 0.005 | -2.776 | -0.633 | 0.007 | -2.577 | -0.588 | 0.012 |
| Frontal_Inf_Oper_R | 2.149 | 0.490 | 0.035 | 2.012 | 0.459 | 0.048 | 1.993 | 0.455 | 0.050 |
| Rolandic_Oper_L | -3.131 | -0.619 | <0.001 | -3.431 | -0.783 | <0.001 | -2.776 | -0.633 | 0.007 |
| Temporal_Inf_L | 2.243 | 0.512 | 0.028 | 2.214 | 0.505 | 0.03 | 1.776 | 0.405 | 0.080 |
| Cingulum_Ant_L | -2.896 | -0.661 | 0.005 | -3.431 | -0.783 | 0.001 | 1.224 | 0.279 | 0.225 |
| Precuneus_L | 2.181 | 0.457 | 0.018 | 2.273 | 0.519 | 0.026 | 2.832 | 0.646 | 0.006 |
| Temporal_Sup_R | -2.610 | -0.595 | 0.011 | -3.219 | -0.697 | <0.001 | 1.911 | 0.436 | 0.060 |
| Temporal_Mid_L | 3.431 | 0.773 | 0.001 | 3.417 | 0.756 | <0.001 | 1.839 | 0.42 | 0.07 |
| Temporal_Mid_R | 2.832 | 0.646 | 0.006 | 2.776 | 0.633 | 0.007 | 3.433 | 0.781 | <0.001 |
| BD *vs.* MDD |  |  |  |  |  |  |  |  |  |
| Frontal_Inf_Tri_L | 3.416 | 0.747 | <0.001 | 3.376 | 0.724 | 0.001 | 3.396 | 0.721 | <0.001 |
| Olfactory_R | 2.571 | 0.562 | 0.012 | 2.874 | 0.628 | 0.005 | 2.964 | 0.648 | 0.004 |
| Supp_Motor_Area_L | -2.639 | -0.574 | 0.013 | -2.720 | -0.595 | 0.008 | -2.11 | -0.462 | 0.038 |
| Rectus_R | -2.318 | -0.507 | 0.023 | -2.301 | -0.503 | 0.024 | -2.603 | -0.570 | 0.011 |
| Frontal_Sup_L | -2.503 | -0.549 | 0.011 | -2.867 | -0.623 | 0.005 | -3.318 | -0.718 | 0.001 |
| Insula_R | -2.887 | -0.632 | 0.005 | -3.195 | -0.699 | 0.002 | -3.416 | -0.728 | <0.001 |
| Cuneus_L | 2.603 | 0.526 | 0.010 | 2.639 | 0.557 | 0.010 | 2.823 | 0.618 | 0.006 |
| Fusiform_R | -2.571 | -0.562 | 0.012 | -2.618 | -0.541 | 0.010 | -2.823 | -0.618 | 0.006 |
| Frontal_Sup_Orb_R | 2.964 | 0.648 | 0.004 | 3.195 | 0.699 | 0.002 | 2.374 | 0.519 | 0.020 |
| Precuneus_L | -3.195 | -0.699 | 0.002 | -2.995 | -0.674 | 0.002 | -3.391 | -0.720 | <0.001 |
| Precuneus_R | -2.589 | -0.523 | 0.010 | -2.647 | -0.577 | 0.010 | -3.320 | -0.719 | <0.001 |
| Temporal_Sup_L | -2.571 | -0.519 | 0.010 | -2.415 | -0.528 | 0.018 | -1.679 | -0.367 | 0.097 |
| Temporal_Sup_R | -3.335 | -0.736 | <0.001 | -3.339 | -0.737 | <0.001 | -1.705 | -0.373 | 0.092 |
| Temporal_Inf_R | 2.209 | 0.483 | 0.030 | 2.438 | 0.533 | 0.017 | 2.224 | 0.486 | 0.029 |
| Paracentral_Lobule_L | 2.268 | 0.496 | 0.026 | 2.133 | 0.467 | 0.036 | 1.353 | 0.296 | 0.18 |

t were calculated from analysis of covariance (ANCOVA) models performed on residualized nodal metrics, after regressing out the effects of age, sex, and years of education. Cohen’ s d values represent standardized effect sizes. All brain regions were defined according to the Automated Anatomical Labeling (AAL) atlas. Abbreviations: t = t-value; Cohen’s d = standardized effect size; R = right; L = left.
