# Supplementary material for: Structural network alterations in adolescent major depression and bipolar disorder: a graph-theoretical and fixel-based analysis
Source: BMC Psychiatry. 2026 Mar 10;26:322. doi: 10.1186/s12888-026-07961-x (PMC13085478; doi:10.1186/s12888-026-07961-x)
Supplement: Supplementary file 3 — Supplementary Material 3: Supplementary Table S2. Differences in FBA measures between patients with MDD and HC [file 12888_2026_7961_MOESM3_ESM.docx]

**Supplementary Table S2. Differences in FBA measures between patients with MDD and HC based on independent-sample T-tests with sex, age covariates.**

| **Tracts** | **FD** | | | **FC** | | | **FDC** | | |
| --- | --- | --- | --- | --- | --- | --- | --- | --- | --- |
|  | **t** | **p** | **Cohen’s d** | **t** | **p** | **Cohen’s d** | **t** | **p** | **Cohen’s d** |
| AF | **-1.738** | **0.036** | **-0.256** | **-1.843** | **0.029** | **-0.298** | **-1.867** | **0.017** | **-0.268** |
| ATR | -0.621 | 0.536 | -0.163 | -0.746 | 0.458 | -0.196 | -0.911 | 0.366 | -0.239 |
| CA | -0.186 | 0.852 | -0.048 | **-0.695** | **0.025** | **-0.185** | -0.433 | 0.188 | -0.114 |
| CC | **-1.645** | **0.027** | **-0.232** | **-3.105** | **0.005** | **-0.391** | **-3.385** | **<0.001** | **-0.401** |
| CG | -0.435 | 0.666 | -0.114 | -0.037 | 0.971 | -0.011 | -0.698 | 0.488 | -0.183 |
| FPT | -0.025 | 0.976 | -0.007 | **-1.224** | **0.036** | **-0.178** | -0.382 | 0.104 | -0.123 |
| FX | 0.541 | 0.593 | 0.142 | -0.919 | 0.362 | -0.241 | -1.057 | 0.295 | 0.178 |
| ICP | -0.891 | 0.377 | -0.234 | **-3.915** | **<0.001** | **-0.496** | **-3.109** | **<0.001** | **-0.365** |
| IFO | 0.264 | 0.796 | 0.068 | **-0.501** | **0.024** | **-0.113** | **-0.848** | **0.017** | **-0.192** |
| ILF | -0.114 | 0.915 | -0.033 | **-1.771** | **0.008** | **-0.245** | **-0.076** | **0.011** | **-0.017** |
| MLF | **-2.798** | **0.021** | **-0.341** | **-1.151** | **0.026** | **-0.145** | **-3.341** | **<0.001** | **-0.387** |
| OR | 0.278 | 0.782 | 0.073 | **-0.862** | **0.041** | **-0.195** | **-0.708** | **0.028** | **-0.160** |
| POPT | -0.056 | 0.956 | -0.015 | **-0.562** | **0.031** | **-0.127** | **-0.13** | **0.022** | **-0.029** |
| SCP | -0.241 | 0.81 | -0.063 | **-2.354** | **0.007** | **-0.301** | **-0.783** | **0.047** | **-0.177** |
| SLF_I | **-3.296** | **0.004** | **-0.413** | -0.514 | 0.609 | -0.135 | **-0.823** | **0.021** | **-0.181** |
| SLF_II | 0.492 | 0.625 | 0.129 | -0.998 | 0.322 | -0.262 | -0.694 | 0.490 | -0.182 |
| SLF_III | **-1.137** | **0.042** | **-0.165** | -0.473 | 0.641 | -0.123 | -0.734 | 0.466 | -0.193 |
| UF | -1.229 | 0.224 | -0.323 | **-1.358** | **0.025** | **-0.184** | -0.706 | 0.483 | -0.185 |

The Cohen’s d quantified the effect size between HC and patients with MDD. The statistics and effect values in bold indicated that the adjusted p-value < 0.05 with FWE-corrected. FD = fiber density; FC = fiber-bundle cross-section; FDC = fiber density and cross-section; abbreviations of the tracts refer to Fig.1.
