# Supplementary material for: Structural network alterations in adolescent major depression and bipolar disorder: a graph-theoretical and fixel-based analysis
Source: BMC Psychiatry. 2026 Mar 10;26:322. doi: 10.1186/s12888-026-07961-x (PMC13085478; doi:10.1186/s12888-026-07961-x)
Supplement: Supplementary file 4 — Supplementary Material 4: Supplementary Table S3. Differences in FBA measures between patients with BD and HC [file 12888_2026_7961_MOESM4_ESM.docx]

**Supplementary Table S3. Differences in FBA measures between patients with BD and HC based on independent-sample T-tests with sex, age covariates.**

| **Tracts** | **FD** | | | **FC** | | | **FDC** | | |
| --- | --- | --- | --- | --- | --- | --- | --- | --- | --- |
|  | **t** | **p** | **Cohen’s d** | **t** | **p** | **Cohen’s d** | **t** | **p** | **Cohen’s d** |
| AF | 0.438 | 0.663 | 0.115 | -0.255 | 0.923 | -0.058 | -0.037 | 0.970 | -0.010 |
| ATR | -0.633 | 0.529 | -0.166 | -0.587 | 0.419 | -0.133 | -0.502 | 0.618 | -0.132 |
| CA | 0.063 | 0.950 | 0.017 | -0.076 | 0.299 | -0.017 | -0.945 | 0.348 | -0.248 |
| CC | -0.298 | 0.767 | -0.078 | **-1.708** | **0.018** | **-0.264** | -0.711 | 0.480 | -0.187 |
| CG | -0.737 | 0.464 | -0.194 | -0.033 | 0.974 | -0.009 | 1.094 | 0.279 | 0.287 |
| FPT | -0.575 | 0.567 | -0.151 | -0.154 | 0.878 | -0.041 | -0.707 | 0.482 | -0.186 |
| FX | 0.152 | 0.880 | 0.040 | **-2.131** | **0.022** | **-0.269** | -0.069 | 0.945 | -0.018 |
| ICP | 0.483 | 0.631 | 0.127 | -0.24 | 0.811 | -0.063 | -0.727 | 0.470 | -0.191 |
| IFO | -0.178 | 0.859 | -0.047 | -0.448 | 0.655 | -0.118 | 0.657 | 0.514 | 0.172 |
| ILF | 0.73 | 0.469 | 0.192 | -0.605 | 0.548 | -0.159 | -0.479 | 0.634 | -0.126 |
| MLF | 0.166 | 0.869 | 0.044 | **-1.911** | **0.019** | **-0.216** | -0.037 | 0.970 | -0.010 |
| OR | -0.053 | 0.958 | -0.014 | -0.973 | 0.335 | -0.255 | 0.655 | 0.515 | 0.172 |
| POPT | -0.883 | 0.381 | -0.232 | 0.176 | 0.861 | 0.046 | 0.444 | 0.658 | 0.117 |
| SCP | 1.05 | 0.298 | 0.276 | -0.032 | 0.975 | -0.008 | -0.224 | 0.823 | -0.059 |
| SLF_I | 0.235 | 0.815 | 0.062 | -0.965 | 0.338 | -0.254 | -0.424 | 0.673 | -0.111 |
| SLF_II | 0.857 | 0.395 | 0.225 | -0.04 | 0.968 | -0.01 | -0.885 | 0.380 | -0.232 |
| SLF_III | **2.569** | **0.012** | **0.301** | -0.093 | 0.926 | -0.024 | 0.715 | 0.478 | 0.188 |
| UF | -0.303 | 0.763 | -0.080 | 0.533 | 0.596 | 0.140 | 0.330 | 0.743 | 0.087 |

The Cohen’s d quantified the effect size between HC and patients with BD. The statistics and effect values in bold indicated that the adjusted *p*-value < 0.05 with FWE-corrected. FD = fiber density; FC = fiber-bundle cross-section; FDC = fiber density and cross-section; abbreviations of the tracts refer to Fig.1.
