# Supplementary material for: Structural network alterations in adolescent major depression and bipolar disorder: a graph-theoretical and fixel-based analysis
Source: BMC Psychiatry. 2026 Mar 10;26:322. doi: 10.1186/s12888-026-07961-x (PMC13085478; doi:10.1186/s12888-026-07961-x)
Supplement: Supplementary file 5 — Supplementary Material 5: Supplementary Table S4. Differences in FBA measures between patients with MDD and BD [file 12888_2026_7961_MOESM5_ESM.docx]

**Supplementary Table S4. Differences in FBA measures between patients with MDD and BD based on independent-sample T-tests with sex, age covariates.**

| **Tracts** | **FD** | | | **FC** | | | **FDC** | | |
| --- | --- | --- | --- | --- | --- | --- | --- | --- | --- |
|  | **t** | **p** | **Cohen’s d** | **t** | **p** | **Cohen’s d** | **t** | **p** | **Cohen’s d** |
| AF | **1.887** | **0.019** | **0.233** | 0.056 | 0.956 | 0.015 | 0.294 | 0.77 | 0.077 |
| ATR | 0.066 | 0.315 | 0.015 | 0.339 | 0.736 | 0.089 | 0.472 | 0.639 | 0.124 |
| CA | 0.208 | 0.210 | 0.047 | 0.105 | 0.917 | 0.028 | 0.71 | 0.481 | 0.186 |
| CC | **2.101** | **0.017** | **0.283** | 0.239 | 0.812 | 0.063 | 0.196 | 0.846 | 0.051 |
| CG | 0.426 | 0.124 | 0.096 | 0.836 | 0.407 | 0.220 | 0.221 | 0.826 | 0.058 |
| FPT | 0.568 | 0.231 | 0.129 | 0.157 | 0.876 | 0.041 | 0.316 | 0.753 | 0.083 |
| FX | **2.208** | **0.011** | **0.307** | **1.833** | **0.039** | **0.249** | 0.868 | 0.389 | 0.228 |
| ICP | 0.714 | 0.478 | 0.188 | 0.754 | 0.454 | 0.198 | 0.961 | 0.34 | 0.252 |
| IFO | 0.378 | 0.707 | 0.099 | 0.467 | 0.642 | 0.123 | 0.193 | 0.848 | 0.051 |
| ILF | 0.62 | 0.538 | 0.163 | 0.788 | 0.434 | 0.207 | 0.043 | 0.966 | 0.011 |
| MLF | **3.259** | **0.005** | **0.428** | 0.425 | 0.672 | 0.112 | 0.294 | 0.77 | 0.077 |
| OR | **0.72** | **0.021** | **0.163** | 1.082 | 0.284 | 0.284 | 0.472 | 0.639 | 0.124 |
| POPT | **0.018** | **0.024** | **0.102** | 0.196 | 0.845 | 0.051 | 0.71 | 0.481 | 0.186 |
| SCP | 0.749 | 0.457 | 0.197 | 0.599 | 0.552 | 0.157 | 0.196 | 0.846 | 0.051 |
| SLF_I | 0.445 | 0.658 | 0.117 | 0.743 | 0.462 | 0.194 | 0.221 | 0.826 | 0.058 |
| SLF_II | 0.3 | 0.765 | 0.079 | 0.704 | 0.484 | 0.185 | 0.521 | 0.605 | 0.137 |
| SLF_III | **1.989** | **0.015** | **0.274** | 0.133 | 0.895 | 0.035 | 0.467 | 0.642 | 0.123 |
| UF | 0.593 | 0.558 | 0.155 | 0.691 | 0.493 | 0.181 | 0.785 | 0.436 | 0.206 |

The Cohen’s d quantified the effect size between patients with MDD and patients with BD. The statistics and effect values in bold indicated that the adjusted *p*-value < 0.05 with FWE-corrected. FD = fiber density; FC = fiber-bundle cross-section; FDC = fiber density and cross-section; abbreviations of the tracts refer to Fig.1.
