# Supplementary material for: Structural network alterations in adolescent major depression and bipolar disorder: a graph-theoretical and fixel-based analysis
Source: BMC Psychiatry. 2026 Mar 10;26:322. doi: 10.1186/s12888-026-07961-x (PMC13085478; doi:10.1186/s12888-026-07961-x)
Supplement: Supplementary file 6 — Supplementary Material 6: Supplementary Table S5. Network-level nodal metrics in the DMN, SN, and CEN [file 12888_2026_7961_MOESM6_ESM.docx]

**Supplementary Table S5. Subnetwork-level averages of nodal metrics in the DMN, SN, and CEN.**

| **Metric** | **HC** | **MDD** | **BD** | **ANCOVA P** | **HC vs MDD P** | **HC vs BD P** | **MDD vs BD P** |
| --- | --- | --- | --- | --- | --- | --- | --- |
| DMN_Efficiency | 0.6706 | 0.6746 | 0.6823 | <0.001 | <0.001 | <0.001 | <0.001 |
| DMN_Degree | 32.5803 | 33.1210 | 34.2643 | <0.001 | 0.002 | <0.001 | <0.001 |
| DMN_Betweenness | 19.7698 | 19.2494 | 19.1251 | 0.003 | 0.007 | 0.001 | 0.497 |
| SN_Efficiency | 0.7064 | 0.7156 | 0.7205 | <0.001 | <0.001 | <0.001 | <0.001 |
| SN_Degree | 37.7889 | 39.6245 | 40.3344 | <0.001 | <0.001 | <0.001 | 0.005 |
| SN_Betweenness | 23.2252 | 24.9737 | 22.1178 | <0.001 | <0.001 | 0.223 | <0.001 |
| CEN_Efficiency | 0.7089 | 0.7142 | 0.7224 | <0.001 | <0.001 | <0.001 | <0.001 |
| CEN_Degree | 37.5891 | 38.5415 | 39.9517 | <0.001 | <0.001 | <0.001 | <0.001 |
| CEN_Betweenness | 34.0714 | 31.8027 | 30.5204 | <0.001 | <0.001 | <0.001 | <0.001 |

Adjusted means from ANCOVA controlling for age, sex, and years of education are reported for HC, MDD, and BD. Post-hoc pairwise comparisons were performed using estimated marginal means. P values < 0.001 are reported as < 0.001. Abbreviations: DMN = default mode network; SN = salience network; CEN = central executive network.
